# Clinical Use of Non-Suture Silk-Containing Products: A Systematic Review

**DOI:** 10.3390/biomimetics8010045

**Published:** 2023-01-18

**Authors:** Jose A. Foppiani, Allan Weidman, Angelica Hernandez Alvarez, Lauren Valentine, Karthika Devi, David L. Kaplan, Samuel J. Lin

**Affiliations:** 1 Division of Plastic Surgery, Beth Israel Deaconess Medical Center, Boston, MA 02215, USA; 2 Harvard Medical School, Boston, MA 022155566, USA; 3 Sri Manakula Vinayagar Medical College, Pondicherry University, Puducherry 605107, India; 4 Department of Biomedical Engineering, Tufts University, Medford, MA 02155, USA

**Keywords:** silk, systematic review, bioinspiration, biomimetics, silk biomaterials, biomimetics in tissue regeneration, silk-derived, clinical utility, biopolymers

## Abstract

Aims: The purpose of this systematic review is to determine how various innovative non-suture silk and silk-containing products are being used in clinical practice, and compare patient outcomes following their use. Methods: A systematic review of PubMed, Web of Science, and Cochrane was completed. A qualitative synthesis of all included studies was then performed. Results: Our electronic search identified 868 silk-related publications, which yielded 32 studies for full-text review. After exclusion, nine studies from 2011 to 2018 were included for qualitative analysis. A total of 346 patients were included which consisted of 37 males and 309 females. The mean age range was between 18–79 years old. The follow-up among studies ranged between one to twenty-nine months. Three studies addressed the application of silk in wound dressings, one on the topical application of silk-derived products, one on silk-derived scaffold in breast reconstruction, and three on silk underwear as adjunct for the treatment of gynecological conditions. All studies showed good outcomes alone or in comparison to controls. Conclusion: This systematic review concludes that silk products’ structural, immune, and wound-healing modulating properties are advantageous clinical assets. Nevertheless, more studies are needed to strengthen and establish the benefit of those products.

## 1. Introduction

Silk has been used reliably in surgery since as early as the second century [1]. Derived from the silk moth, *Bombyx mori*, its structure is comprised of fibroin fiber and a glue-like protein, sericin [2]. These two components provide silk with the unique characteristics that make it available as a biomedical material. Fibroin allows for the creation of porous 3D structures, including sponges, foams, and scaffolds. The protein structure of sericin contains many beta sheets which afford high mechanical strength [3]. Given these characteristics, silk has been used effectively as a suture material in surgery and represents a promising target of biomaterials research with applications throughout medicine.

Future goals for the use of silk biomaterials are vast and include many applications that are already undergoing research testing. For example, silk has been proposed as an innovative option to overcome the current limitations of surgical fixation devices [4]. Fixation devices such as screws, plates, and K-wires made from silk would be absorbable and biocompatible, as well as offer therapeutic benefits such as invoking a minimal inflammatory response and even promoting bone remodeling [5]. Silk, among other bioabsorbable materials, represents a frontier in the field of surgery that will continue to be the focus of intense investigation.

Currently, silk is most often used clinically in the care of wounds. Silk has been shown to possess excellent cutaneous wound-healing properties through the promotion of cell attachment, migration, and collagen deposition [6]. Further, silk can be impregnated with growth factors, antimicrobials, and extracellular matrix derivatives, which have been shown to improve graft healing and collagen production [7]. Given these properties among many others, silk products or mixtures containing the components of silk have been utilized to repair skin [8], abdominal wall, liver [9,10], vascular tissue [11], nerves, and components of intervertebral discs [12] in different models.

Despite significant advancements in the field of biomaterials, there is currently no comprehensive review of patient outcomes following the use of silk products. With research efforts underway to expand the use of silk in medicine, it is crucial to understand how similar products are already being used and with what effect. With this knowledge, it would be possible to better guide ongoing basic science research to produce effective and safe products. The purpose of this systematic review of the literature is to determine how innovative non-suture silk products are being used in the clinical practice of medicine and compare patient outcomes following their use.

## 2. Methods

**Figure 1:**
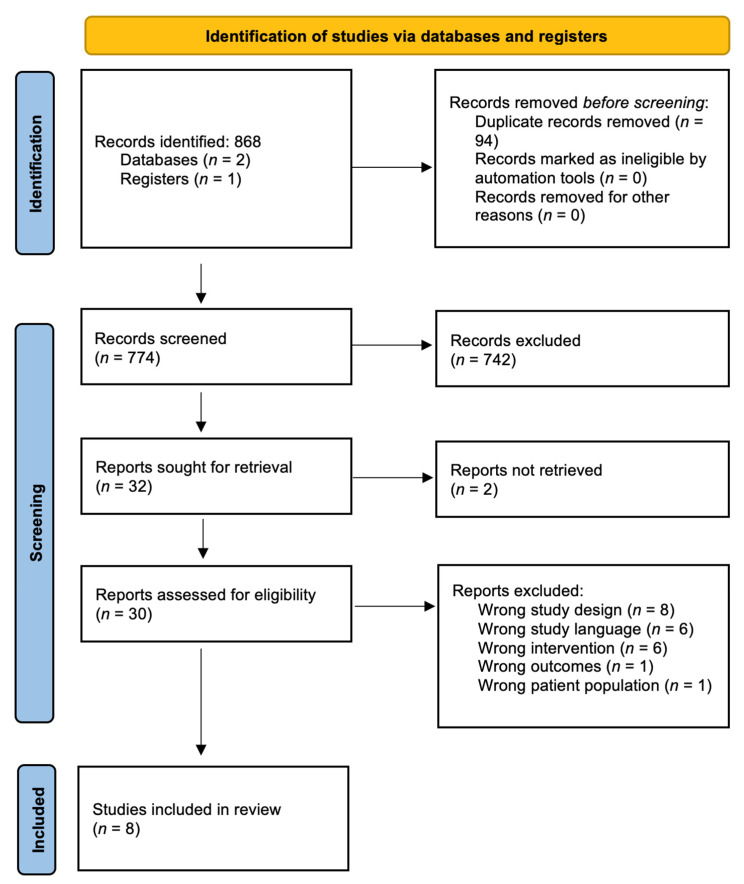
Systematic Reviews and Meta-analysis (PRISMA) guidelines flow diagram.

This study protocol was prospectively registered with PROSPERO (Study#: CRD42022367931). Completion of the study was performed in accordance with the Preferred Reporting Items for Systematic Reviews and Meta-Analysis (PRISMA) statement guidelines (Figure 1) [13].

### 2.1. Eligibility Criteria

Criteria for included studies were defined as any adult patients that received treatment involving a non-suture silk product. Use of silk products as suture material were excluded due to the vast and established research on the topic. The full eligibility criteria are accessible at PROSPERO and are as followed:

Inclusion criteria:

- Male and female patients who underwent a clinical intervention with silk or silk-based products.
- Observational studies and clinical trials
- Studies in English, French, and Spanish

Exclusion criteria:

- Editorials, commentary reports, abstracts, and letters to the editors
- Pediatric population (less than 18 years of age)
- Animal studies
- Ongoing studies
- Cadaveric studies
- Sutures made of or containing silk-derived products

### 2.2. Search Strategy

A comprehensive research review using subject headings, controlled vocabulary, and keywords was conducted on 25 September 2022, on MEDLINE (in Ovid), Web of Science, and the Cochrane Central Register for studies published until 2022. A second search was conducted on 26 November 2022, in the same databases to capture any newly published or missed studies. Our full-text search strategy is accessible at PROSPERO.

### 2.3. Study Selection

The search results were uploaded into the online systematic review program Covidence to conduct study selection [14]. Five independent reviewers performed a two-stage screening process for study selection (J.A.F., A.H.A., L.V., A.W., and K.D.). First, titles and abstracts were screened. A third reviewer then moderated if discordances were present and resolved any conflicts. Next a full-text analysis was performed by the same five reviewers. If conflicts arose between reviewers, a third reviewer moderated a discussion to come to a joint decision.

### 2.4. Data Extraction/Synthesis

Data extraction was guided by a predetermined checklist: first author last name, year of publication, type of device/material, number of patients, gender, age, time to follow up, protocol defining the use of the device, and patient health outcomes following intervention with the device/material.

### 2.5. Outcomes

These results of this systematic review focus on the outcomes following the use of silk-derived products or tissue in their respective populations, specifically highlighting therapeutic successes, objective and subjective assessment of outcomes, and complications.

### 2.6. Quality Assessment

To assess the risk of bias, the National Institute of Health (NIH) quality assessment tool was utilized [15]. Each article was categorized as follows: “low risk,” “moderate risk,” or “high risk” of bias. Seven studies were found to have a low risk of bias while one had a moderate risk. Details of study characteristics are found in Table 1.

### 2.7. Statistical Analysis

Due to the heterogenicity of the topics covered in the studies constituting this systematic review, it was not possible to perform any analysis beyond a qualitative synthesis.

## 3. Results and Discussion

The electronic search initially identified 868 silk-related publications, which ultimately yielded 32 studies for full-text review (Figure 1). After thorough assessment and subsequent exclusion, eight studies from 2011 to 2018 were included for qualitative analysis (Table 1) [16,17,18,19,20,21,22,23,24]. These consisted of two case control studies and six randomized clinical trials.

A total of 346 patients were included, comprising 37 men and 309 women. The age range was 18 to 79 years old. The follow-up among studies ranged from one to twenty-nine months. Studies were organized into four categories based on their clinical application: management of gynecologic conditions, wound dressings, cosmetic utility, and breast reconstruction. Three studies investigated the use of silk undergarments for adjunct treatment of various gynecologic conditions including Corazza et al. [21] for lichen simplex chronicus, D’Antuono et al. 2011 [23] for lichen sclerosus, and D’Antuono et al. 2012 [22] for recurrent vulvovaginal candidiasis. Schulz et al. [19], Napavichayanum et al. [18], and Hasatsri et al. [17] addressed applications in wound dressings. Berardesca et al. [16] reported their development of a facial topical application used as a cosmetic measure, while Karp et al. [24] reported on the use of a silk supporting scaffold in breast reconstruction. A complete summary of reported outcomes can be found in Table 2.

### 3.1. Gynecologic Conditions

#### 3.1.1. Results

Three randomized clinical trials assessed the use of Dermasilk briefs for the adjunctive treatment of various gynecologic conditions, which included vulvar lichen simplex chronicus (VLSC), vulvovaginal candidosis, and vulvar lichen sclerosis [21,22,23]. Dermasilk fabric consists of 100% pure sericin-free fibroin that is combined with AEM 5772/5, an antimicrobial agent [21]. Corazza et al. [21] explored the use of Dermasilk underwear in comparison to cotton underwear in the management VLSC. Twenty women with VLSC were enrolled and were all initially managed in an open-label active treatment phase with topical 0.1% mometasone furoate (MMF) for one week, then randomized to use briefs made of Dermasilk or 100% cotton. The use of silk fabric briefs yielded a lower number of mean monthly corticosteroids applications, longer symptom-free intervals, and more favorable global objective scores (GOS) and global subjective scores (GOS). In comparison, participants using cotton briefs demonstrated mean symptom values that were worse compared to those at the end of their active treatment phase. Overall, they concluded that silk fabric briefs were more efficacious than cotton briefs in controlling itching (*p* = 0.013; t test), burning (*p* = 0.174; Mann–Whitney U test), stinging (*p* = 0.081; Mann–Whitney U test), GSS (*p* = 0.030; t test), and GOS (*p* = 0.294; t test) after corticosteroid treatment [21].

D’Antuono et al. 2012 and D’Antuono et al. 2011 investigated the use of the same Dermasilk underwear as an adjuvant tool in managing recurrent vulvovaginal candidiasis (VC) and vulvar lichen sclerosus (VLS), respectively [22,23]. In both studies, researchers conducted a double-blind randomized control trial where patients were either assigned Dermasilk or cotton briefs for six months. In the 2011 D’Antuono et al. study assessing efficacy for management of VLS, patients were treated with clobetasol propionate 0.05% ointment (CPO) and a vitamin E-containing moisturizer for six months and randomized into wearing either 100% cotton or Dermasilk briefs [23]. Regarding symptomatic management, relief of burning, soreness and itching showed statistically significant improvements in the Dermasilk group. These same patients also experienced significant reductions in erythema and improvements in the speed of symptom reduction at one month. The authors therefore concluded that the adjunctive use of Dermasilk briefs appeared to offer symptomatic relief and clinical improvement for patients being managed with VLS [23].

In the 2012 study assessing the management of VC, patients were treated with fluconazole weekly for six months in addition to either Dermasilk or cotton briefs [22]. The results had a temporal trend to them, with both groups initially experiencing similar symptom reduction after one month but increasingly diverging in favor of the Dermasilk group at three months. After six months, there was a statistically significant decrease in itching, erythema, and burning in the patients wearing Dermasilk. An additional significant decrease of VC recurrence was found in the Dermasilk group, with only 32 out of 48 patients (66.7%) having no or one recurrence while 29 out of 48 patients (60.5%) who wore cotton experienced two or more recurrences. Resultantly, the authors concluded that adjunctive use of Dermasilk briefs could aid in symptom reduction and limit infection recurrence when managing candidiasis [23].

#### 3.1.2. Discussion

Silk-derived products have shown promising results when participating in the management of gynecologic conditions such as lichen sclerosus, vulvar lichen simplex chronicus, and vulvovaginal candidiasis [21,22,23]. In addition to each of the standard of care therapies such as a corticosteroid or antimycotic agents, the auxiliary use of Dermasilk briefs consistently demonstrated symptom reduction and clinical improvement for all three conditions. This correlates with other research specifically directed toward silk application in gynecologic conditions, with Cook et al. conducting a systematic review of treatment in recurrent VC and revealing that adjunct use of Dermasilk briefs showed a trend toward achieving resolution and better symptomatic relief compared to placebo [25].

The first property of silk that makes it advantageous for gynecologic conditions is its antimicrobial utility. At different points during its production, it is possible to manipulate various points and create structurally unique and adaptable forms of the polymer. These include adjustments of the molecular weight and concentration of beta sheets [26]. Additionally, silk-based biomaterials are capable of being loaded with antibiotics or antimycotics which are then subsequently released into the surrounding tissue. Controlled impregnation and sustained release of antibiotics from silk biomaterials have been demonstrated, highlighted in a paper by Pritchard et al. where antibiotics were loaded into various forms of silk, including films, hydrogels, and microspheres. With in vivo use of a murine infected wound model, these drugs were continually released and successfully suppressed bacterial growth better than a comparison group of antibiotics in solution [26]. In the three papers referenced above, the active antimicrobial product was AEM 5772-5, a stable quaternary ammonium compound that works by damaging the cell wall of microorganisms and subsequently inducing cellular lysis [22]. An additional benefit to this antimicrobial finish is that it covalently binds to silk fiber, thus fixing it permanently and not allowing it to detach with repetitive uses or washes [23]. This antimicrobial property successfully resulted in a decrease in recurrence of vulvovaginal candidosis when impregnated into silk briefs when compared to cotton, thus highlighting how this property of silk lends itself well to infection control.

Another property of silk to highlight with regards to gynecologic conditions is its ability to decrease vulvar irritation and friction due to its fiber structure. Silk has long, cylindrical-shaped filaments that collectively form a smooth surface, ultimately forming a product that is both non-irritating for the skin as well as comfortable for patient use [22]. The interaction of silk products with the inflamed, irritated surface of the skin has been previously detailed in the literature. Zheng et al. detailed how the silk structure can be manipulated by adjusting the molecular weight to form a smaller, narrower structure that is ultimately smoother [27]. This lends well to inflammatory conditions of the skin, as demonstrated by the Dermasilk briefs which led to improvement in symptoms such as itching, burning, and erythema for patients with VC, VLSC, and VLS. In all three studies, this was compared to cotton, a tight, synthetic fabric with a rougher texture, which cannot offer the same smooth surface and can even ultimately exacerbate the irritation of the skin. Other studies have capitalized on this essentially frictionless property of silk. While excluded from our search strategy, Koller et al. revealed in their cohort of 22 children that the non-irritating properties of silk were also applicable for the treatment of atopic dermatitis [28]. Similarly, in their study, Schaunig et al., showed that those same properties also extended to chronic inflammatory conditions such acne vulgaris [29]. Therefore, this cylindrical shape of the filaments leads to a smooth surface, ultimately decreasing friction against the already-inflamed mucosa in these various conditions and facilitating healing.

In addition to the antimicrobial and anti-irritative properties that silk offers, another important property that is highlighted in clinical utility is its moisture-absorbing ability. As emphasized by D’Antuono et al., the structure of silk very closely resembles the stratum corneum of skin. Given that the stratum corneum is the outermost barrier layer, silk thus mimics many favorable properties that the human skin exhibits [22]. This is emphasized by the fact that silk fibroin has been previously engineered in various clinical applications to play a role in the creation of artificial skin [30]. For these various vulvovaginal conditions, the skin pH and local microbiota of the tissue must be carefully regulated in order to facilitate healing rather than disease progression. Opportunistic yeast such as *Candida albicans* grow and thrive in conditions that are warm and moist [31]. Given that silk can absorb a significant amount of moisture without dampening the material itself, it adequately regulates the balance of humidity and temperature of the vulvar skin, limiting its proliferation [23]. Resultantly, patients who used Dermasilk briefs had long-term symptomatic benefits and significantly fewer recurrences than those who wore cotton briefs. Overall, the silk offers advantageous heat-regulating and hygroscopic properties that ultimately provide an environment that limits and prevents infection.

### 3.2. Silk Applications in Wound Dressings

#### 3.2.1. Results

Schulz et al. [19] evaluated a silk dressing versus a nylon mesh covered by porcine type 1 collagen (Biobrane); split-thickness skin graft (STSG) donor sites were used as wound base. The authors studied subjective and objective measurements of scar evaluation. In the subjective assessment, Vancouver Scar Scale (VSS) and Patient and Observer Scar Assessment Scale (POSAS) scores were used. Despite a subjective enhancement in the quality of both scars and a high satisfaction rate following the treatments being reported in their populations, neither intervention yielded a statistically significant difference between VSS and POSAS scores. Using the VSS, both dressings were reported to have a scar pigmentation equal to untreated skin or slightly hypopigmented. Vascularity and pliability were assessed as normal. The objective evaluation of scar formation using laser Doppler spectrometry found significant differences solely in scar perfusion (SO_2_; *p* = 0.012). Data showed a lower SO_2_ in both areas that were treated compared with healthy skin. Lastly, the only difference between treatments was cost; the price of Biobrane was approximately 10 times higher than Dressilk (per 1 cm^2^) in the treatment center.

Napavichayanum et al. [18] studied a previously developed bacterial cellulose wound dressing containing silk serine versus an antiseptic paraffin gauze dressing with chlorhexidine acetate at 0.5% known as Bactigras^®^. The authors used blinded randomization to assign the arm participants. They evaluated wound-healing time and wound quality of STSG donor sites. There were no statistically significant differences in healing time for both dressings. Wound quality was assessed using erythema level, melanin levels, transepidermal water loss (TEWL) levels, and the VSS. The levels of erythema and melanin of both dressings were significantly lower when compared to normal skin at healing time and at one month. At three months, the skin treated with either of the dressings showed similar pigmentation to normal skin as measured by the melanin levels. After six months, the erythema levels of the areas treated with the dressings were still higher than normal skin. Areas treated with bacterial cellulose wound dressings containing silk sericin and PHMB (BCSP) were significantly lower than Bactigras^®^-treated wounds. TEWL levels using both dressings, representing the quantity of water that is lost from the skin and reflect the barrier function of the skin, were at all times significantly higher than normal skin. Nevertheless, the Bactigras^®^ group showed significantly higher TEWL level at one, three, and six months compared to the BCSP group. Lastly, VSS was used to assess the scar with four parameters: vascularity, pigmentation, pliability, and height. Overall VSS scores of both dressings were not significantly different.

Hasatsri et al. [17] describe a novel silk fibroin-based bilayered wound dressing developed by the authors that was compared to Bactigras^®^. In this randomized study, the wound dressing’s adhesiveness, clinical safety, and efficacy were assessed. This new dressing was, when compared to Bactigras^®^, removed from the wound bed with greater ease. Furthermore, while no cells adhered to the novel dressing, a significant number of cells were found adherent to the Bactigras ^®^ dressing (∼21 × 104 cells/dressing) indicating that Bactigras^®^ damages some of the new epithelial cells upon removal. With regards to safety, 95.9% of the bilayered wound dressing volunteers and 98.6% of the Bactigras^®^ volunteers showed no evidence of any adverse effect on the application sites. In terms of efficacy, the donor sites treated with the bilayered wound dressing (11 ± 6 days) were fully healed significantly faster than those treated with Bactigras^®^ (14 ± 6 days). Lastly, the TEWL index of the donor sites treated with the bilayered wound dressing was significantly lower than those treated with Bactigras^®^. This infers a faster TEWL recovery of the sites treated with the bilayered wound dressing.

#### 3.2.2. Discussion

The care of wounds has been a focus of mankind since its origin, with basic principles of wound healing first described and applied at least 4000 years ago [32]. An important aspect of modern wound care is the application of carefully selected dressings [33,34]. Those dressings generally function as a barrier to environmental bacteria and support the healing processes by providing the wound bed with the optimum environment [35]. Many of the properties that make silk advantageous for use in gynecologic conditions translate to wound healing as well, which include the antimicrobial, anti-irritative, and hygroscopic properties of the material. As mentioned before, fibroin is a mimicker of the stratum corneum and offers barrier protection for healing wounds.

This systematic review showed that new wound dressing options containing silk material had a relatively shorter healing time, less pigmentation, and better aesthetic outcomes in their respective cohorts compared to Bactigras^®^ and Biobrane [17,18,19]. Interestingly, TEWL levels in all dressings were significantly higher than normal adjacent, uninjured skin [17,18,19]. Bactigras^®^ has been established as a core dressing in burn patients to treat donor sites of split thickness skin grafts [36,37]. Muangman et al., in their randomized control trials with 16 patients using Bactigras^®^ and 16 Telfa AMD (Polyhexamethylene Biguanide), found that while the use Bactigras^®^ had good outcomes, one of its drawbacks was the damage its removal caused to new epithelium during dressing changes [38]. Newly formed epithelium is fragile, and any break in this barrier can result in infection or delayed wound healing. One of the advantages of the silk-derived dressings highlighted in this review is that they are non-adhesive in nature, thus changing these dressings does not result in trauma to the underlying wound bed or newly formed epithelium [17,18,19]. Overall, while effective wound dressings are commercially available, silk-derived dressings may enhance wound care by offering improved cosmetic outcomes and optimized adhesive properties to protect at-risk areas.

An additional factor to consider when determining the material to use for wound dressings is cost. Silk sericin is a natural by-product that is found in various settings, including in agricultural waste and wastewater from textile factories [18]. Because of its disposable nature, the cost of acquiring silk sericin is relatively cheap. The overall cost of BCSP production was low at around 0.21 USD, which is increased to approximately 0.29 USD when factoring in other manufacturing costs. In comparison, the cost of an equivalent-sized piece of Bactigras is approximately 0.46 USD, and the cost of collagen-based dressings is significantly higher at around 19 USD. Therefore, the use of silk products may also offer a cost advantage due to competitive manufacturing pricing when compared to other standard of care dressings.

### 3.3. Topical Application of Silk-Containing Complex for Cosmesis

#### 3.3.1. Results

In a randomized, double-blind, vehicle-controlled study by Berardesca et al. [16], Gold Silk Sericin (GSS) was combined with niacinamide and signaline to form a cosmetically active complex. A prior washout of the skin was followed by the applications of the GSS complex plus a vehicle, consisting of a simple oil-in-water emulsion, versus the vehicle alone after subject randomization. A variety of facial skin aging endpoints were then investigated. Treatment with the vehicle + GSS promoted at week 4 (*p* < 0.01) and week 8 (*p* < 0.001) a significantly greater increase in terms of stratum corneum hydration versus the vehicle alone. Likewise, at week 4 (*p* < 0.05) and week 8 (*p* < 0.0001) a greater decrease in TEWL was driven by the novel complex. Treatment with the complex at both weeks 4 and 8 (*p* < 0.05) promoted a significantly greater increase in gross elasticity a well as net elasticity compared to vehicle alone. Also, treatment with the complex promoted greater improvements in smoothness and wrinkling, as well as uniformity and skin tone when compared with vehicle alone. Additionally, when week 8 was reached, improvement for all the previously mentioned parameters was found to be statistically significant (vehicle+ GSS versus vehicle alone, *p* < 0.05).

#### 3.3.2. Discussion

In this study, one of the two main components of silk, sericin, was used due to its unique characteristics. Specifically, a particular variety of sericin, Gold Silk Sericin, was included. Gold Silk Sericin is carefully extracted from Irodori silk cocoons by a proprietary process in order to maintain its gold color [16]. Generally, silk-derived sericin has been shown in various studies to be medically useful due to its antioxidant, anti-microbial, anti-aging, moisture-absorbing, and wound-healing properties [36,37]. Further, the non-silk components of the cosmetic complex utilized in this study, niacinamide and signaline, also had significant contributions to the anti-aging properties of the topical agent. Niacinamide, for example, has been shown to prevent photoaging by preventing the loss of dermal collagen and improving the skin’s barrier function. As a result, it has been reported to decrease the aesthetic appearance of photoaging on the face [39,40,41]. Signaline, on the other hand, is derived from olive oil and fatty alcohols in a specific type of wax. As for its utility, it has been reported by its commercial manufacture to augment signal transduction intracellularly [16].

The advantages of this product are clear in the results presented by Berardesca et al. They concluded that their complex can address aged female skin by offering improvements in stratum corneum hydration, barrier function, and elasticity [42]. While it is difficult to attribute these properties to sericin versus niacinamide or signaline, the use of silk-derived products in this case does additionally present several disadvantages. Primarily, the use of a specific variety of sericin, Gold Silk Sericin, limits the generalization of the results presented. Given the novel and proprietary extraction methods utilized, other investigators likely could not reproduce the complex used in this study. For the same reasons, cost may hinder the generalization and overall utility of the complex compared to other anti-aging products. While it was possible this sericin variety was used only for its color, there was little explanation by the authors as to the benefits of this specific variety compared to other, possibly more accessible and cheaper, varieties of sericin.

Anti-aging therapies have seen an increase in focus from the medical research community over the last few decades [43]. While surgical techniques for rejuvenation have been heavily described, both plastic surgery and dermatology have strived to develop non-invasive options for treatment and prevention of facial aging [43,44]. The study by Berardesca et al. showed a significantly greater increase in gross elasticity and net elasticity versus vehicle alone at weeks 4 and 8 as well as improvements in parameters associated with wrinkling (volume), smoothness, and uniformity [16]. In comparison, Manosroi et al. showed that 20 patients treated with Oryza Sativa Linn, a nanosome-loaded glutinous rice extract, experienced increased skin hydration, skin elastic extension, and skin elastic recovery of +48.73%, −24.51%, and +35.98% respectively [45]. While this study highlights the potential of other naturally occurring compounds and proteins, this new study on another agent for anti-aging is but one of many. The anti-aging properties of silk-derived products thus would need to be tested against other products to define their place among emerging anti-aging products.

### 3.4. Silk Scaffold in Breast Reconstruction

#### 3.4.1. Results

Lastly, in their study, Karp et al. [24] conducted a multicenter prospective study in patients undergoing a two-stage breast reconstruction using the SERI surgical scaffold. The female patients included in this study initially received a subpectoral tissue expander followed by an exchange with a breast implant. Data was collected at various time points including the first postoperative visit, the visits for tissue expansion, the second stage surgery to replace tissue expanders with permanent implants, as well as follow-up visits at six, twelve, eighteen, and twenty-four months after surgery. The mean (95% CI) satisfaction score in the patient population was 9.6/10 (9.44–9.67). The mean investigator satisfaction at the second stage surgery and after the two-year follow-up was reported as 9.3/10. During the second stage surgery, surgeons observed local tissue growth through the SERI device; capsule adherence to the tissue expander and integration with the tissue expander indicated that SERI scaffold did not interfere with normal healing. Indeed, the majority of scaffolds (98.7%) were fully integrated with the surrounding tissue and had vascularized capsules (96.8%). In term of safety profile, skin or tissue necrosis was reported in thirteen breasts (8.1%), wrinkling or rippling in thirteen breasts (8.1%), seroma in eight breasts (5.0%), wound dehiscence in eight breasts (5.0%), and capsular contracture in four breasts (2.5%). Additionally, the cohort of Karp et al. experienced one unilateral periprosthetic infection as well as two cases of unilateral mastitis. Overall, the SERI scaffold showed high and consistent levels of subject and investigator satisfaction while demonstrating it to be safe and to provide adequate soft-tissue stability in the lower breast through two years of follow-up.

#### 3.4.2. Discussion

In this study, a silk-derived surgical scaffold notable for its bioresorbability was used in breast reconstruction [24]. This product derives a large portion of its unique properties from the silk protein, fibroin. Fibroin is most often used as a suture material as a result of its highly hydrophobic and therefore minimally resorbable nature [46]. However, it is possible to alter fibroin’s chemistry in order to allow for resorption and integration into human tissue. This is accomplished through support of cell attachment, synthesis of collagen, and promotion of tissue development [47]. As a result, it is possible to achieve predictable long term bioresorption through manipulation of the protein’s bioresorption rate. Further, fibroin can be engineered for complex control of mechanical properties like strength and stiffness [46]. Ultimately, these unique and modifiable properties allow for the creation of a robust surgical mesh or scaffold that is biocompatible with evidence of a mild and self-limiting foreign body response.

Consequently, prior evidence from an abdominal wall reconstruction study has shown that the use of a surgical mesh of the same material diminished foreign body complications given its ability to be resorbed and replaced by host tissue [48]. Interestingly, testing of the mechanical properties of the mesh over time demonstrated similar results as those expected of the musculature of the abdominal wall. This supports the expectation that the mesh becomes integrated into host tissue. As a result, the silk-derived mesh can avoid complications associated with restriction of abdominal wall mobility, which may be seen in non-resorbable mesh [48]. Use of the mesh also subsequently reduced risk for bacterial infection by promoting macrophage involvement as it is resorbed. This offers a unique advantage to other commonly used meshes, which have had limited success long-term when used in contaminated surgical environments [49]. While these benefits were shown primarily in reconstruction of the abdominal wall, this study by Karp et al., among others, demonstrates similar advantages when used in breast reconstruction.

With regard to patient outcomes, the use by Karp et al. of a silk-derived breast scaffold led to several complications. However, the prevalence of these complications, including skin necrosis, seroma, capsular contracture, and infection, were relatively low [24,50]. The previously mentioned study by Horan et al.,, utilizing the SeriFascia surgical mesh, had similarly positive outcomes, with no serious adverse events associated with the mesh reported by postoperative day 94 [48]. Further, a letter to the editor described the use of SERI surgical scaffold by 28 surgeons in 16 different types of procedures, including those involving the breast. Out of 141 patients who underwent surgeries with the use of the silk-derived scaffold, only three device-related adverse events were reported. Specific to the procedures of the breast (not including flap-based operations), 103 patients received the silk-derived scaffold and only one experienced a complication [50]. Therefore, while still in need of further randomized and long-term studies, silk-derived scaffolds are seemingly safe and offer a wide variety of advantages given their biocompatibility.

### 3.5. Limitations

While this is an original study to systematically review and compare the outcomes and complications of the clinical use of silk-derived products, it has its own limitations. Given the heterogeneity between studies and their clinical uses, it was difficult to compare outcomes and prevented us from performing to complete a meta-analysis of the results. Additionally, our results yielded a female predominance (309/346, 89%) given that multiple included studies involved the use of silk-derived products for breast and gynecologic conditions. The studies included in the review often did not stratify patient outcomes by age or only provided a range of ages of patients, making it difficult to assess outcome differences by age. Regardless of these limitations, the silk-based products included in this study demonstrate the versatility of silk’s anti-inflammatory properties in the treatment of chronic inflammatory conditions, but also demonstrate the need for further investigation studies to establish its place in current treatment regimens.

## 4. Conclusions

This systematic review is the first study to investigate the clinical use of non-suture, silk-derived products and silk-containing products in the adult population. Silk, silk-derived products, and silk-containing products have been thoroughly investigated in the pre-clinical and experimental literature. However, this study demonstrates that the translation of those studies into clinical practice is lagging. The results of this systematic review establish that while there are currently few clinical studies on non-suture silk-derived products, the studies published highlight how silk has shown versatility through its applications as anti-aging products, scaffolds, adjunct undergarments, and wound dressings. Overall, the main conclusion to be drawn from the current literature is that silk products have shown promise, but a translation of pre-clinical and animal studies into clinical studies is needed to establish their place in patient care.

## Figures and Tables

**Table 1:** Study Characteristics.

Title	Author, Year	Intervention/ Device	Total Patients (N)	Sex		Age (µ)	Follow Up (Month)
				Male	Female		
Long-Term Scar Quality After Treatment Of Standardized Partial-Thickness Skin Graft Donor Sites	Schulz, 2018	Dressilk	11	8	3	51	29
		Biobrane					
Inflammatory Reaction, Clinical Efficacy, and Safety of Bacterial Cellulose Wound Dressing Containing Silk Sericin and Polyhexamethylene Biguanide for Wound Treatment	Napavichayanun, 2018	Bacterial cellulose wound Dressings containing silk sericin and PHMB (BCSP)	21	16	5	60	6
		Bactigras					
Randomized Clinical Trial of the Innovative Bilayered Wound Dressing Made of Silk and Gelatin: Safety and Efficacy Tests Using a Split-Thickness Skin Graft Mode	Hasatsri, 2015	Silk fibroin-based bilayered wound dressing	23	13	10	37.3	13
		Bactigras					
Randomized, Double Blinded, Vehicle Controlled, Split-Face Study to Evaluate the Effects of Topical Application of a Gold Silk Sericin/Niacinamide/Signaline Complex on Biophysical Parameters Related to Skin Aging	Berardesca, 2015	Gold Silk Sericin Complex + Oil in Water (Vehicle)	30	0	30	40–70	2
		Oil in Water (Vehicle)					
Effectiveness of Silk Fabric Underwear as an Adjuvant Tool in the Management of Vulvar Lichen Simplex Chronicus: Results of a Double-Blind Randomized Controlled Trial.	Corazza, 2015	Silk fabric underwear (Dermasilk)	20	0	20	n/a *	1
		Cotton fabric underwear					
Use of Dermasilk Briefs in Recurrent Vulvovaginal Candidosis: Safety and Effectiveness	D’Antuono, 2012	Silk fabric underwear (Dermasilk)	96	0	96	30.25	6
		Cotton fabric underwear					
Dermasilk Briefs in Vulvar Lichen Sclerosus: An Adjuvant Tool.	D’Antuono 2011	Silk fabric underwear (Dermasilk)	42	0	42	51.5 (median)	6
		Cotton fabric underwear					
Seri Surgical Scaffold in 2-Stage Breast Reconstruction: 2-Year Data from a Prospective, Multicenter Trial.	Karp 2017	SERI surgical scaffold	103	0	103	50.7	24

* n/a—not applicable.

**Table 2:** Study protocols and reported outcomes.

Title	Details on Device/Intervention	Protocol	Outcomes	Nih Quality Assessment
Schulz, 2018	Dressilk: fibroin based dressing	2 adjacent partial-thickness skin graft donor sites were treated with Biobrane or Dressilk.	All patients reported a high satisfaction and improved scar quality following the intervention. No statistically significant differences between VSS and POSAS scores were reported.	Low Risk of Bias
	Biobrane: nylon mesh covered by porcine type 1 collagen			
Napavichayanun, 2018	BCSP: Acetobacter xylinum was used for cellulose production which was then sterilized using gamma ray (25 kGy)	Single-blinded, randomized controlled study where STSG donor site wound were created with an electrical dermatome. Then, it was immediately covered with the 1:1000 adrenaline gauze. BCSP or Bactigras^®^ (control) were then used as primary dressing.	For both dressings wound-healing time was around 19 ± 5 days. The levels of melanin and erythema for BCSP-treated wounds were significantly lower than the wounds treated with the control and scar quality was reported to be higher in the BCSP group	Moderate Risk of Bias
	Bactigras^®^: medicated chlorhexidine paraffin gauze dressing			
Hasatsri, 2015	A combination of silk fibroin, silk sericin, and gelatin was used to prepare the wound dressing material which was then gamma irradiated	Single-blinded, randomized controlled study where STSG donor site wounds were divided into equal halves, randomly assigned to receive either the bilayered wound dressing or Bactigras.	Bactigras was more adhesive than silk dressing leading to epithelial damage during removal. On the day of donor site healing, the Median TEWL of the area treated with Bactigras and the bilayer wound dressing were respectively 2.8 ± 0.8 and 2.3 ± 0.9 times higher than that of the adjacent skin on the first day.	Low Risk of Bias
	Bactigras^®^: medicated chlorhexidine paraffin gauze dressing			
Berardesca, 2015	GSS is a cosmetically active complex obtained from irodori cocoons that is combined with niacinamide and Signaline.	Before the start of the trial a 4 weeks wash-out period was implemented. The patients applied the Vehicle and the Vehicle plus GSS complex to either side of the face twice daily at a rate of 2 mg cm2, following the assigned randomization.	Vehicle+ GSS yielded improvement in wrinkling (volume), smoothness, hydration, elasticity, and uniformity compared to vehicle alone.	Low Risk of Bias
	Simple oil-in-water emulsion (vehicle)			
Corazza, 2015	Briefs consisting of 100% pure sericin-free fibroin impregnated with antimicrobial protection (AEM5772/5)	Following 1 week of topical 0.1% mometasone furoate (MMF) ointment applications, participants entered a 4-week double-blind maintenance phase. They were randomized to wear either silk fabric or cotton briefs.	Following the corticosteroid treatment silk fabric briefs were more efficacious than cotton briefs in controlling itching (*p* = 0.013), burning (*p* = 0.174), stinging (*p* = 0.081), global subjective score (*p* = 0.030), and global objective score (*p* = 0.294)	Low Risk of Bias
	Briefs made of 100% cotton.			
D’antuono, 2012	Briefs consisting of 100% pure sericin-free fibroin impregnated with antimicrobial protection (AEM5772/5)	6 months of fluconazole treatments with 150 mg weekly in addition to randomly assigned Dermasilk or control briefs to wear day and night for the duration of the treatment.	Following 6 months of treatment, the patients in the Dermasilk group showed a significantly greater decrease in itching, erythema, and burning compared to the patients using cotton briefs. Most patients in the Dermasilk group had 0 to 1 recurrence of vulvovaginal candidiasis, whereas, in the cotton group, most had two or more recurrences.	Low Risk of Bias
	Briefs made of 100% cotton.			
Karp 2017	SERI Surgical Scaffold: bioresorbable silk-derived scaffold for soft-tissue support.	A single-arm study which enrolled women who underwent 2 stage breast reconstruction. SERI was applied during stage 1 of the 2 stage reconstruction.	SERI was associated with a high degree of patient and investigator satisfaction. A majority of scaffolds were integrated with the surrounding tissue and had vascularized capsules. Tissue or skin necrosis occurred in 13 breasts (8.1%), wrinkling/rippling in 13 (8.1%), seroma in 8 (5.0%), wound dehiscence in 8 (5.0%), and capsular contracture in 4 (2.5%). Three breast infections were reported.	Low Risk of Bias
D’antuono 2011	Briefs consisting of 100% pure sericin-free fibroin impregnated with antimicrobial protection (AEM5772/5)	Following a 2-week period of washout treatment with a combination of a vitamin E moisturizer and clobetasol propionate 0.05% ointment was initiated. Patients were advised to apply half a fingertip unit (0.25 g) of clobetasol propionate 0.05% ointment every night and the same dose of moisturizer every morning for 6 months in addition to the randomly assigned briefs.	A statistically significant improvement in burning, erythema, and soreness was reported in the Dermasilk group when compared to the control group. Additionally, the severity of itching and fissures trended in favor of the Dermasilk group.	Low Risk of Bias
	Briefs made of 100% cotton.			

Bacterial cellulose wound dressings containing silk sericin and PHMB (BCSP), split-thickness skin graft (STSG), transepidermal water loss (TEWL), Patient and Observer Scar Assessment Scale (POSAS), and (f) the Vancouver Scar Scale (VSS), Gold Silk Sericin (GSS), vulvar lichen simplex chronicus (VLSC), and DS (Dermasilk).

## Data Availability

Data sharing not applicable. No new data were created or analyzed in this study. Data sharing is not applicable to this article. All information relevant to this systematic review is part of the manuscript, figures, tables, and/or digital supplemental content. Additional information can be found within the publicly available PROSPERO protocol for this study. If any further information is required, the reader may contact the corresponding author for clarifications.
